# Supplementary material for: Rice Serine Hydroxymethyltransferases: Evolution, Subcellular Localization, Function and Perspectives
Source: Plants (Basel). 2024 Apr 16;13(8):1116. doi: 10.3390/plants13081116 (PMC11053755; doi:10.3390/plants13081116)
Supplement: Supplementary file 1 [file plants-13-01116-s001.zip › Supplemental Figure.pdf]

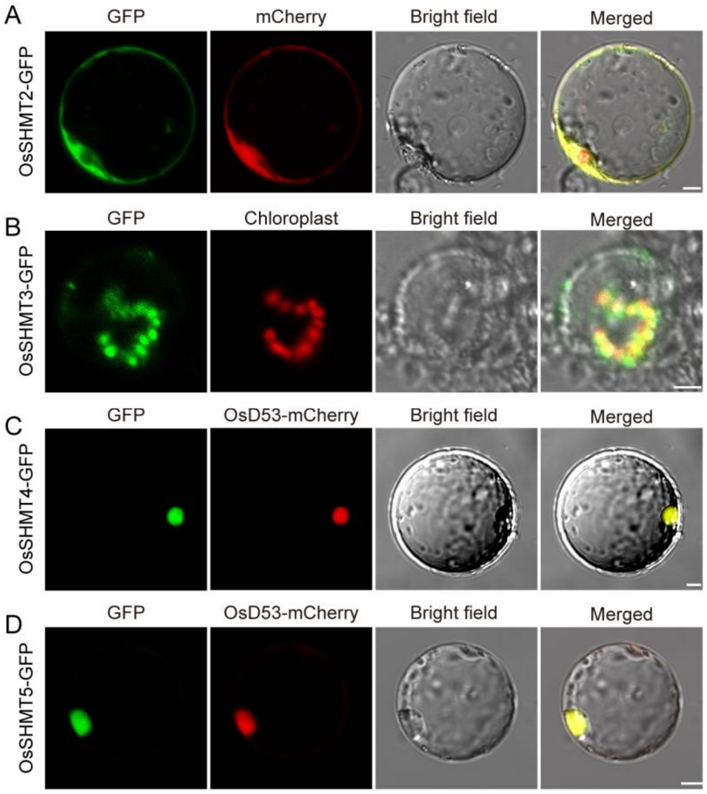

Figure S1. Subcellular localization of OsSHMTs in rice protoplast. (A-D) Confocal microscopic images showing the subcellular localization of OsSHMTs. mCherry serves as a cytoplasmic marker. Chloroplast autofluorescence is utilized as a marker for chloroplasts. OsD53-mCherry functions as a nuclear marker. Bars = 5 μm.

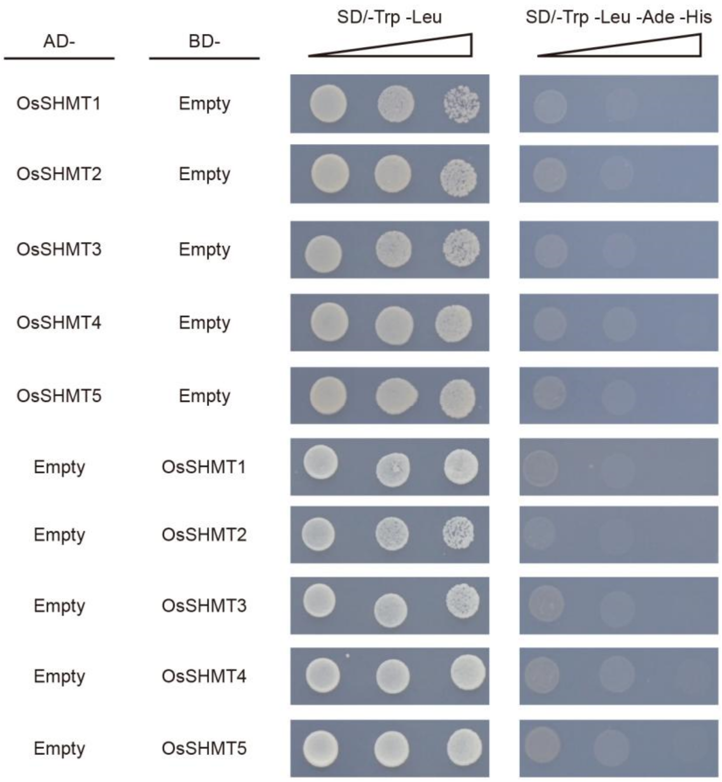

Figure S2. Negative controls for the Y2H assay. DDO, SD/-Trp/-Leu; QDO, SD/-Trp/-Leu/-Ade/-His. AD, fused with activation domain; BD, fused with binding domain.
